# Genetic Screening for *TLR7* Variants in Young and Previously Healthy Men With Severe COVID-19

**DOI:** 10.3389/fimmu.2021.719115

**Published:** 2021-07-23

**Authors:** Xavier Solanich, Gardenia Vargas-Parra, Caspar I. van der Made, Annet Simons, Janneke Schuurs-Hoeijmakers, Arnau Antolí, Jesús del Valle, Gemma Rocamora-Blanch, Fernando Setién, Manel Esteller, Simon V. van Reijmersdal, Antoni Riera-Mestre, Joan Sabater-Riera, Gabriel Capellá, Frank L. van de Veerdonk, Ben van der Hoven, Xavier Corbella, Alexander Hoischen, Conxi Lázaro

**Affiliations:** ^1^ Department of Internal Medicine, Hospital Universitari de Bellvitge, Bellvitge Biomedical Research Institute (IDIBELL), L’Hospitalet de Llobregat, Barcelona, Spain; ^2^ Hereditary Cancer Program, Catalan Institute of Oncology, Program in Molecular Mechanisms and Experimental Therapy in Oncology (Oncobell), Bellvitge Biomedical Research Institute (IDIBELL), L’Hospitalet de Llobregat, Barcelona, Spain; ^3^ Centro de Investigación Biomédica en Red de Cáncer (CIBERONC), Madrid, Spain; ^4^ Department of Human Genetics, Radboud University Medical Center, Nijmegen, Netherlands; ^5^ Department of Internal Medicine and Radboud Center for Infectious Diseases (RCI), Radboud Institute for Molecular Life Sciences (RIMLS), Radboud University Medical Center, Nijmegen, Netherlands; ^6^ Radboud Expertise Center for Immunodeficiency and Autoinflammation and Radboud Center for Infectious Disease (RCI), Radboud University Medical Center, Nijmegen, Netherlands; ^7^ Josep Carreras Leukaemia Research Institute (IJC), Badalona, Spain; ^8^ Institucio Catalana de Recerca i Estudis Avançats (ICREA), Barcelona, Spain; ^9^ Physiological Sciences Department, School of Medicine and Health Sciences, University of Barcelona (UB), Barcelona, Spain; ^10^ Faculty of Medicine and Health Sciences, Universitat de Barcelona, Barcelona, Spain; ^11^ Department of Intensive Care, Hospital Universitari de Bellvitge, Bellvitge Biomedical Research Institute (IDIBELL), L’Hospitalet de Llobregat, Barcelona, Spain; ^12^ Department of Intensive Care, Erasmus MC, Rotterdam, Netherlands; ^13^ School of Medicine, Universitat Internacional de Catalunya, Barcelona, Spain

**Keywords:** COVID-19, SARS-CoV-2, host genetics, TLR7, immunodeficiency, genetic screening

## Abstract

**Introduction:**

Loss-of-function *TLR7* variants have been recently reported in a small number of males to underlie strong predisposition to severe COVID-19. We aimed to determine the presence of these rare variants in young men with severe COVID-19.

**Methods:**

We prospectively studied males between 18 and 50 years-old without predisposing comorbidities that required at least high-flow nasal oxygen to treat COVID-19. The coding region of *TLR7* was sequenced to assess the presence of potentially deleterious variants.

**Results:**

*TLR7* missense variants were identified in two out of 14 patients (14.3%). Overall, the median age was 38 (IQR 30-45) years. Both variants were not previously reported in population control databases and were predicted to be damaging by *in silico* predictors. In a 30-year-old patient a maternally inherited variant [c.644A>G; p.(Asn215Ser)] was identified, co-segregating in his 27-year-old brother who also contracted severe COVID-19. A second variant [c.2797T>C; p.(Trp933Arg)] was found in a 28-year-old patient, co-segregating in his 24-year-old brother who developed mild COVID-19. Functional testing of this variant revealed decreased type I and II interferon responses in peripheral mononuclear blood cells upon stimulation with the TLR7 agonist imiquimod, confirming a loss-of-function effect.

**Conclusions:**

This study supports a rationale for the genetic screening for *TLR7* variants in young men with severe COVID-19 in the absence of other relevant risk factors. A diagnosis of TLR7 deficiency could not only inform on treatment options for the patient, but also enables pre-symptomatic testing of at-risk male relatives with the possibility of instituting early preventive and therapeutic interventions.

## Introduction

A proportion of patients with COVID-19 develop fatal lung injury and multi-organ failure due to systemic host-immune inflammatory processes triggered by the viral infection (1). Advanced age, male sex and chronic disease such as diabetes and obesity are common in patients with more severe forms of COVID-19 (2–4). However, these risk factors cannot explain why critical disease also occurs in young (below 50 years of age) and apparently healthy individuals.

In the past months, several publications have identified loci and genes associated with COVID-19 susceptibility for severe COVID-19 by using comprehensive GWAS studies or genome, exome or candidate gene analyses (5). However, most of these susceptibility alleles showed risk values too low (odds ratio <2) to be regarded as predictive genomic markers. Some of the loci reported include *ABO* blood group (6, 7), *ACE2* (8), *TMPRSS2* (8), several HLA alleles (5, 9), *APOE* (10), and *IFITM3* (11). On the other hand, rare variants in genes encoding for members of the type I/III interferon (IFN) pathway showed a higher estimated risk to severe COVID-19 (12, 13).

In July 2020 rare, deleterious germline variants in the X-chromosomal Toll-like receptor 7 (*TLR7)* gene were reported in young and, otherwise, healthy males with severe COVID-19. In these two brother pairs, rapid whole-exome sequencing identified both a maternally inherited 4-nucleotide deletion [c.2129_2132del; p.(Gln710Argfs*18)] and a missense variant [c.2383G>T; p.(Val795Phe)]. Both variants were associated with impaired type I and II IFN responses upon stimulation with the TLR7 agonist imiquimod, supporting the importance of intact TLR7 signaling in COVID-19 pathogenesis (13). Most recently these findings were replicated in an independent Italian cohort study of males <60 years of age with severe COVID-19 (79 severe cases versus 77 control cases), showing that 2.1% of severely affected males harbored deleterious *TLR7* variants compared to none of the asymptomatic participants. These variants were demonstrated to decrease transcription of type I and II IFN-related genes in patient peripheral blood mononuclear cells (PBMC) treated with imiquimod, supporting a loss of function effect (14).

In follow-up of the recent discovery of TLR7 deficiency in patients with severe COVID-19, we aimed to prospectively determine the presence of *TLR7* variants in young and previously healthy men with severe COVID-19.

## Methods

This is a joint study performed at the Hospital Universitari de Bellvitge – IDIBELL, L’Hospitalet de Llobregat, Barcelona, Spain; and the Radboud University Medical Center, Nijmegen, The Netherlands and the Erasmus Medical Center, Rotterdam, The Netherlands.

### The Barcelona Cases

From March to July 2020, researchers from Hospital Universitari de Bellvitge - IDIBELL prospectively collected biological samples from young patients without comorbidities related to severe COVID-19. Selection criteria were: 1) patients aged between 18 and 50 years old; 2) absence of known comorbidities associated with most severe forms of COVID-19 (BMI ≥ 30kg/m2, diabetes mellitus, hypertension, chronic heart, pulmonary or kidney disease, or an immunocompromised state); and 3) SARS-CoV-2 related lung injury requiring high flow oxygen devices or mechanical ventilation. Ten male patients fulfilled these selection criteria (**Table 1**). Eight patients (patients 1-8) were also evaluated by the COVID Human Genetic Effort, although no pathogenic variants were identified in any of the 13 type I IFN pathway genes studied (12).

**Table 1:** Demographic and clinical findings of investigated patients.

Patient	Sequencing	Gender	Age (y)	Ethnicity	Comorbidities	ARDS^a^	ICU	ECMO
1	Sanger	M	31	Caucasian	no	yes	no	no
2	Sanger	M	44	Caucasian	no	yes	yes	no
3	Sanger	M	41	Latin (Venezuela)	no	yes	yes	yes
4	Sanger	M	40	Caucasian	no	yes	yes	no
5	Sanger	M	50	Latin (Peru)	no	yes	yes	no
6	Sanger	M	48	Caucasian	no	yes	yes	no
7	Sanger	M	45	Latin (Peru)	no	yes	yes	no
8	Sanger	M	31	Latin (Honduras)	no	yes	yes	no
9	Sanger	M	47	Latin (Peru)	no	yes	no	no
10^b^	Sanger	M	30	Latin (Dominican Republic)	no	yes	yes	no
11	Sanger	M	21	Caucasian	no	yes	yes	no
12	Sanger	M	36	Caucasian	no	yes	yes	no
13^b^	WES	M	28	Caucasian	no	yes	yes	no
14	WES	M	31	Caucasian	no	yes	no	no

ARDS, acute respiratory distress syndrome; ECMO, extracorporeal membrane oxygenation; ICU, intensive care unit; F, female; M, male; WES, whole-exome sequencing; Y, years.

a ARDS Definition Task Force. Acute Respiratory Distress Syndrome - The Berlin Definition. JAMA. 2012;307(23):2526-2533. doi:10.1001/jama.2012.5669.

b Patients 10 and 13 resulted carriers of TLR7 variants and belong to family 1 and 2, respectively.

Informed consent was obtained from all patients and relatives, and the IDIBELL Research Ethics Committee approved this study (PR152/20). Demographic, epidemiological, laboratory and clinical data were collected. Treatments specifically used to treat COVID-19 at any time during admission were also documented.

DNA was isolated from total blood either using a Maxwell instrument RSC (Promega, Madison, WI, USA) or QIAGEN Flexigene DNA kit (Qiagen, Germany). Nine PCR primer pairs (Sigma-Aldrich, MO, USA) were designed to cover the whole coding region of *TLR7* (HGNC ID:15631). PCR was performed using DreamTaq MasterMix (ThermoFisher Scientific, Waltham, MA, USA), products were purified using EXO-SAP (New England Biolabs) and sequenced using the BigDye Terminator v.3.1 Sequencing Kit (Applied Biosystems, CA, USA) in an ABI Prism 3730 XL Genetic Analyzer (Applied Biosystems CA, USA). Primers and PCR conditions are available upon request. Mutation Surveyor software was used to detect variants and nomenclature was given according to HGVS guidelines. All variants identified were submitted to Alamut Software Suite v2.15.0 (Interactive Biosoftware) to retrieve population frequency and *in silico* prediction data.

### The Dutch Cases

At the Radboud University Medical Center in Nijmegen and the Erasmus Medical Center in Rotterdam, the Netherlands, patients were screened prospectively in a clinical setting from December 2020 to February 2021 with the following criteria: 1) males between 18 and 40 years of age; 2) absence of comorbidities known to be associated with severe COVID-19 (BMI ≥ 30kg/m2, diabetes mellitus, hypertension, chronic heart, pulmonary or kidney disease, or an immunocompromised state); and 3) PCR-confirmed SARS-CoV-2 infection requiring high-flow oxygen therapy or ICU admission. A total of 4 patients (patients 11-14) fulfilled these inclusion criteria and underwent clinical Sanger sequencing (patients 11 and 12) or rapid whole-exome sequencing (patient 13 and 14) to specifically assess genetic variants in *TLR7* (**Table 1**). Written informed consent was obtained from patient 13 whose clinical data has been included in this study. Rapid whole-exome sequencing was performed similar to previous reports (13). Sanger sequencing was done according to standard diagnostic procedures at the Department of Human Genetics, Radboud University Medical Center, protocols and primers sequences are available upon request.

For patient 13, in whom a rare *TLR7* missense variant was identified, functional testing was performed to evaluate impaired type I and II IFN responses due to *TLR7* loss-of-function, as described previously (13). In brief, venous blood was drawn and collected in EDTA tubes (Monject). Subsequently, peripheral mononuclear blood cells (PBMC) were isolated by density centrifugation of blood, diluted 1:1 in pyrogen-free saline over Ficoll-Paque (Pharmacia Biotech). Cells were washed twice in saline and resuspended in cell culture medium (Roswell Park Memorial Institute [RPMI] 1640, Gibco) supplemented with gentamicin, 10 mg/mL; L-glutamine, 10 mM; and pyruvate, 10 mM. PBMC were then stimulated at 5 × 10^5^ cells/well in round-bottom 96-wells plates (Greiner) for either 4 hours (for transcription of type I IFN genes) and 7 days [for production of the type II IFN, (IFNγ)] in the presence of 10% human pool serum at 37°C and 5% carbon dioxide, in two separate experiments. Apart from a negative medium (RPMI) control, the TLR7 agonist imiquimod (imidazoquinoline compound, Invivogen) was used at a concentration of 5 μg/mL. After the 4 hour incubation period, the supernatants were discarded and the remaining cell pellets were resuspended in RLT buffer (Qiagen) and snap frozen to be stored at −80°C until processing for RNA isolation. In addition, after the 7 day incubation and a centrifugation step, supernatants were collected and stored at −20°C until measured using enzyme-linked immunosorbent assay in case of the 7 day timepoint.

## Results

### *TLR7* Sequencing Results

**Figure 1:**
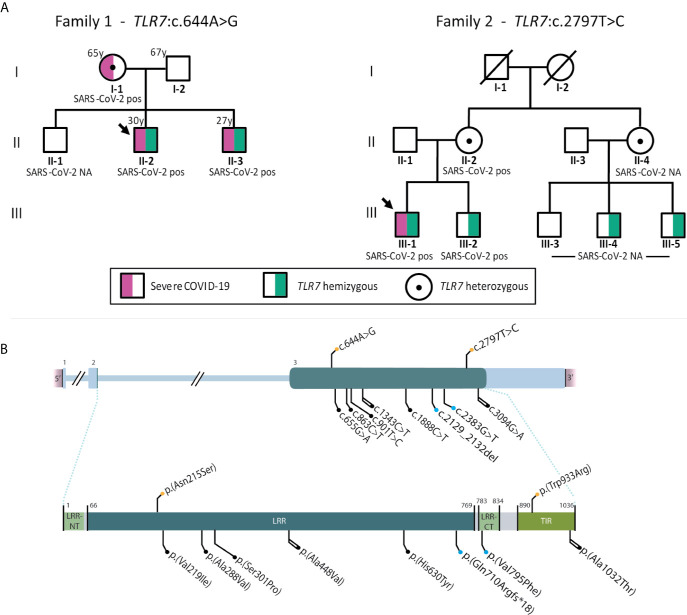
**(A)** displays pedigrees of families 1 (patient 10) and 2 (patient 13) with segregation analysis. **(B)** shows schematic representation of *TLR7* (HGNC ID:15631) variants reported to date in severely affected COVID-19 cases. Variants in cDNA (top) and protein (bottom). Color code: orange, variants found in the present series; blue, previously reported in van der Made (13); black, previously reported in Fallerini (14). Shape code: circle, missense variants; square, frameshift variants. Line code: single, reported in one case; double, reported in 2 cases.

A total of 14 patients were included, with a median age of 38 (IQR 30-45) years. Putative deleterious *TLR7* missense variants were identified in two patients (patient 10 and 13). Both variants, c.644A>G; p.(Asn215Ser) in patient 10 and c.2797T>C; p.(Trp933Arg) in patient 13, were not previously reported in our in-house databases nor in the population database gnomAD (15). The p.(Asn215Ser) variant affects a highly conserved nucleotide and amino acid in the *TLR7* leucine-rich region domain and is predicted damaging or possibly damaging by *in silico* software (**Table S1**). The p.(Trp933Arg) variant is also located at an evolutionarily highly conserved position within the TIR domain, important for downstream signaling *via* adapter proteins, and is considered deleterious by *in silico* effect predictors (**Table S1**). These *TLR7* variants and other previously reported variants in COVID-19 patients are shown schematically in **Figure 1**.

### Patients’ Characteristics

Patient 10 (family 1, proband) was a 30-year-old man from Latin origin (Dominican Republic) with no general risk factors predisposing to severe COVID-19. He developed pneumonia with bilateral consolidations on a computed tomography (CT) scan and fulfilled the criteria of acute respiratory distress syndrome (ARDS) secondary to PCR-proven COVID-19 (**Table 2**). The patient received antiviral treatment with remdesivir and immunosuppressive therapy with dexamethasone. Due to respiratory insufficiency, the patient was intubated and admitted in ICU. The patient was successfully extubated after 4 days of mechanical ventilation and was discharged from ICU after 6 days. After the identification of the *TLR7*:p.(Asn215Ser) variant in the patient, segregation analysis confirmed co-segregation in both his brother and mother, in hemi- and heterozygous state, respectively (**Figure 1**). The eldest brother lives in the Dominican Republic and was therefore not available for testing. According to relatives, there is no evidence that he has been infected with SARS-CoV-2. The youngest brother had no previous medical history but also contracted severe COVID-19, requiring mechanical ventilation and ICU admission at another hospital (**Table 2**). These brothers therefore represent the third brother pair with severe COVID-19, following the initial report (13). The 65 year-old mother suffered from obesity, dyslipidemia, type 2 diabetes and hypertension, and was also admitted in ICU due to critical respiratory failure caused by COVID-19. She was discharged from the ICU 16 days after admission. Main demographic, clinical, laboratory, and radiological findings of the three relatives are summarized in **Table 2**.

**Table 2:** Demographic, clinical, laboratory, and radiological findings of investigated patients.

	Family 1 *TLR7*:c.644A>G			Family 2 *TLR7*:c.2797T>C	Reference ranges
	Proband	Brother	Mother	Proband	
**Demographic characteristics**					
Date of hospitalization	July/2020	March/2020	August/2020	January/2021	
Age, y	30	27	65	28	
Sex	Male	Male	Female	Male	
Medical history	None	None	Obesity, dyslipidemia, hypertension, type 2 diabetes,	Vasovagal syncope	
**Clinical characteristics at presentation**					
Time from symptom onset to hospitalization, d	7	6	12	2	
Symptoms at disease onset	Dyspnea, cough, fever, myalgia	Dyspnea, cough, fever, headache	Dyspnea, cough, fever, myalgia	Dyspnea, cough, fever, respiratory arrest	
Imaging features (CT scan)	Bilateral pulmonary consolidations	Bilateral pulmonary consolidations	Bilateral pulmonary consolidations	Multiple ground glass opacities and consolidations in all lung segments	
**ICU admission**					
Time from symptom onset to ICU admission, d	8	7	12	7	
Medical reason for ICU admission	Respiratory insufficiency	Respiratory insufficiency	Respiratory insufficiency	Respiratory failure, respiratory arrest, resuscitation at home.	
Disease severity status on admission, SOFA score^*^	3	3	4	6	
**Laboratory findings at ICU admission**					
Chemistry					
Alanine aminotransferase, U/L	135	14	23.5	41	<40
Albumin, g/L	37	31	28.1	23	35 - 52
Alkaline phosphatase, U/L	131	66	84.0	222	≤ 129
Aspartate aminotransferase, U/L	92	22	73.8	37	≤ 39
Cardiac troponin, high sensitivity, ng/L	NA	7	NA	NA	≤ 13
Creatine kinase, U/L	51	35	180	NA	≤ 189
Creatinine, μmol/L	60	57	57.1	84	44-97
eGFR, mL/min/1.73 m2	>90	>90	>90	>90	>90
γ-Glutamyltransferase, U/L	243	27	34.8	263	≤ 70
Lactate dehydrogenase, U/L	381	432	1088.4	201	<250
Blood count					
Hemoglobin, g/L	112	114	110	121	130 - 165
Lymphocyte count, ×10^9^/L	1.8	1.47	2.19	1.64	1.3-3.4
White blood cell count, ×10^9^/L	18	10.7	14.74	8.4	3.9-9.5
Platelet count, ×10^9^/L	385	408	416	369	149 - 303
Coagulation					
Activated partial thromboplastin time ratio	0.95	0.98	1.00	37	0.8-1.2
D-dimer, ng/mL	<250	463	2400	3660	<250
Fibrinogen, g/L	> 7	> 7	5.5	4,2	2.76-4.71
Prothrombin time ratio	1.38	1.32	1.10	NA	0.8-1.2
Inflammatory markers					
C-reactive protein, mg/L	346.6	267	203.98	196	<3
Ferritin, μg/L	1957.6	920	384.9	845	30 - 400
Procalcitonin, μg/L	0.28	NA	0.1	4.31	<0.5
IL-6, ng/L	48.4	1.5	24	NA	≤ 6.9
Secondary complications	None reported	catheter-related bloodstream infection	None reported	Small ventral pneumothorax at admission after resuscitation at home. Bilateral subsegmental pulmonary embolisms.	
Duration of viral shedding after COVID-19 onset (positive SARS-CoV-2 PCR), d	Positive at admission, no follow-up measurement	Positive at admission, no follow-up measurement	Positive at admission, no follow-up measurement	Positive before admission, PCR negative at day 29	
Duration of ventilatory support, d	4	10	7	24 days (ongoing)	
Duration of ICU stay, d	6	12	16	24 days (ongoing)	
**Follow-up**					
Time from ICU discharge to hospital discharge, d	3	11	5	NA	
Complications during follow-up period	None reported	None reported	None reported	NA	
Treatments	R, D	H, L-R, MP, I, T	R, D, T	D	

COVID-19, coronavirus disease 2019; CT, computed tomography; ICU, intensive care unit; eGFR, estimated glomerular filtration rate; NA, not assessed; PCR, polymerase chain reaction; SARS-CoV-2; severe acute respiratory syndrome coronavirus 2; SOFA, Sequential Organ Failure Assessment.

The treatments were administered to the patients as follows: D, Dexamethasone 8 mg daily for 10 consecutive days; R, Remdesivir 200mg intravenously the first day and 100mg daily the next four days; T, Tocilizumab 600 mg single dose intravenously; L-R, Lopinavir 400mg-Ritonavir 100mg orally twice daily for three days; H, Hydroxychloroquine orally 400mg twice daily the first day and 200mg twice daily the next 10 days; I, Interferon β1b 0.25mg every other day subcutaneously for 3 days.

^*^The SOFA score is calculated using 6 systems: respiratory, coagulation, hepatic, cardiovascular, central nervous, and kidney. Scores range from 0 for normal function to 4 for most abnormal and are summed for a final range of 0 to 24. An initial score of 2 to 3 is associated with 6% mortality; an initial score of 4 to 5 is associated with 20% mortality.

**Figure 2:**
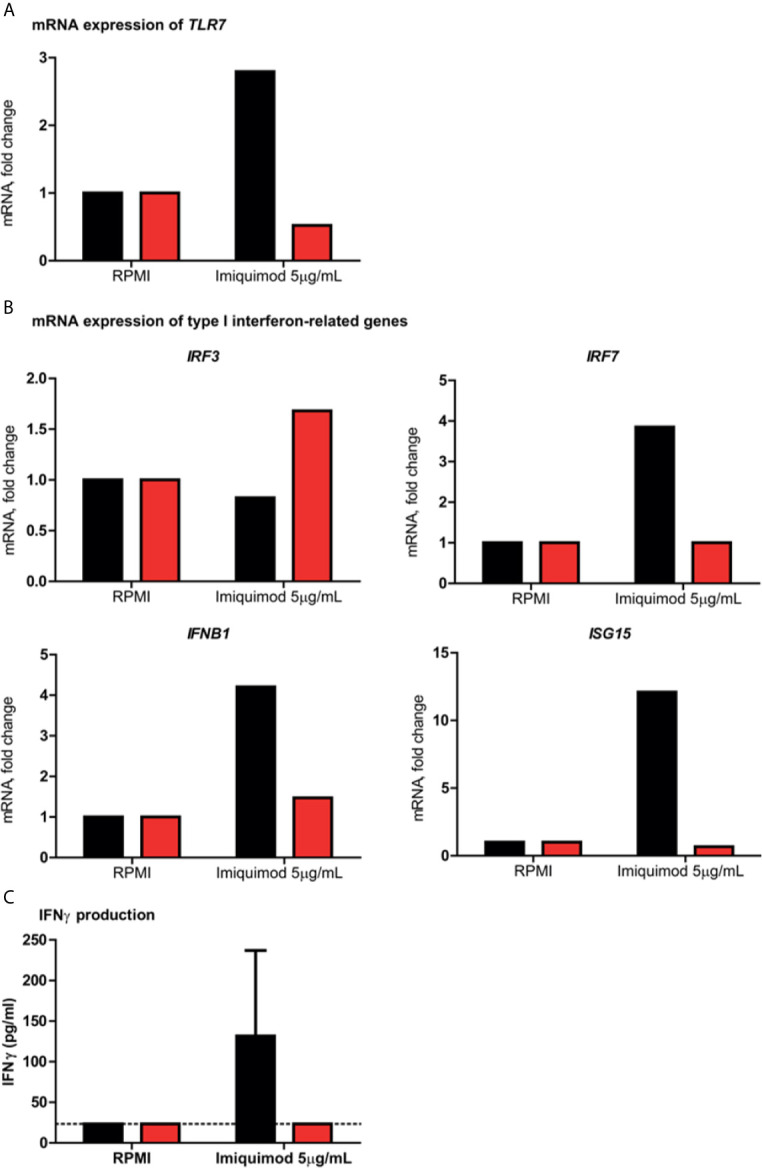
Assessment of *ex vivo/in vitro* type I and II interferon responses in peripheral blood mononuclear cells. In all panels, patient 13 is indicated in red and the healthy controls in black. **(A)** shows the fold change in *TLR7* mRNA expression in patient 13 and a healthy male control after stimulation for 4 hours with the TLR7 agonist imiquimod, compared to the negative medium control RPMI. **(B)** displays the fold change in mRNA expression of type I IFN–related genes *IRF3, IRF7, IFNB1* and *ISG15* induced by imiquimod as compared to RPMI. **(C)** shows the production of IFN-γ production after imiquimod stimulation for 7 days as compared with unstimulated control cells (RPMI) from patient 13 and two healthy male controls. The dotted line indicates the assay’s detection limit. Abbreviations: *IRF3*, interferon regulatory factor 3; *IRF7*, interferon regulatory factor 7; *ISG15*, interferon-stimulated gene 15; and *IFNB1*, interferon beta 1.

Patient 13 (family 2, proband) was a 28-year-old male from Caucasian origin (The Netherlands) without previous medical history or comorbidities. The patient complained of progressive dyspnea and shortness of breath. Following rapid clinical deterioration and respiratory insufficiency, the patient was intubated and hospitalized in ICU at a peripheral hospital. A CT-scan showed multiple diffuse ground-glass opacities and consolidations in all lung segments, meeting the criteria for ARDS. Treatment consisted of mechanical ventilation with prone positioning, intravenous dexamethasone, and the antibiotics ceftriaxone and ciprofloxacin. Despite this therapeutic regiment, the patient’s condition further deteriorated and he was referred to the Erasmus Medical Center for possible ECMO treatment. However, with continuing prone positioning he gradually improved before ECMO was required. A repeated CT scan also showed subsegmental pulmonary embolisms for which intravenous heparin was started. In the following weeks, the patient gradually recovered and was successfully extubated and eventually discharged from the hospital. The patients’ first-degree family contracted COVID-19 at the time the patient developed symptoms, including his 24-year-old brother, who had only minor symptoms but was shown to be a carrier of the *TLR7* variant (**Figure 2B**). Information on the exact SARS-CoV-2 viral titers during infections were unavailable. In addition, two male cousins, sons of a maternal aunt, were proven to be carriers of this variant. These individuals had not contracted (symptomatic) COVID-19. Based on the *TLR7* variant carriership, these individuals were fast-tracked for early vaccination.

### Functional Testing in Primary Immune Cells

In PBMC isolated from patient 13, the type I and II IFN responses were evaluated upon stimulation with the TLR7 agonist imiquimod, in order to assess the functional impact of the p.(Trp933Arg) variant. It was shown that TLR7 stimulation in the patient resulted in a defective upregulation of *TLR7* mRNA expression, as well as that of the type I IFN-related genes involved in the TLR7 pathway *IRF7, IFNB1* and *ISG15*, as compared to a healthy male control (**Figures 2A, B**). Expression of *IRF3*, a transcription factor that is induced by TLR3 and not TLR7, showed a modest increase of almost 1.7 fold in the patient, but was not induced in the healthy control. Moreover, the production of IFNγ upon exposure with imiquimod was deficient in the patient, compared to two healthy male controls (**Figure 2C**).

## Discussion

In this cohort of young males affected with severe COVID-19 rare *TLR7* variants were prospectively identified in 2 out of 14 cases (14.3%). In 10 cases from Spain, one patient was shown to carry a new missense variant [c.644A>G; p.(Asn215Ser)]. The variant was predicted as damaging by *in silico* programs and segregated in a brother who also contracted severe COVID-19. In addition, four Dutch cases were studied, leading to the identification of another patient with a novel, unique missense variant [c.2797T>C; p.(Trp933Arg)] located in the highly conserved TIR domain. Functional analysis of the latter variant demonstrated defective type I and II IFN responses, similar to those documented in the previous reports (13, 14).

A role for the TLR7 receptor has been described in the host defense against single-stranded RNA viruses as well as in the pathogenesis of autoimmune disease such as SLE (16). However, no human mutations in *TLR7* had been reported before the SARS-CoV-2 pandemic. It should be noted that complete TLR7 deficiency is estimated to be extremely rare, because endosomal TLRs (TLR3, TLR7, TLR8, and TLR9) play an essential, non-redundant biological role in host survival (17, 18). Therefore, these rare *TLR7* variants are unlikely to be an explanation for severe COVID-19 in the general population. Distinct from the previously published studies that have identified (enrichment of) rare *TLR7* variants in severe COVID-19 who were 1) males <35 years of age without comorbidities (13), 2) males <60 years of age with or without comorbidities (14) or 3) a group of unselected patients (19), in this study we have selected males <50 years of age without comorbidities predisposing to severe COVID-19. Despite the small cohort, the yield of TLR7 variants was unexpectedly high (14.3%) encouraging that screening for *TLR7* rare variants in severely affected men may be fruitful. Elderly individuals also carry rare, loss of function *TLR7* variants (14), but are more difficult to identify due to other predisposing general risk factors for severe disease in people of advanced age. Therefore, we suggest the following screening criteria: young men (<50 year of age) suffering from severe COVID-19 requiring at least high-flow oxygen therapy, and who were previously healthy. Affected young brother pairs, as well as pedigrees suggestive for X-linked segregation, should be further prioritized.

We furthermore hypothesize that some rare variants compromise essential functional domains in TLR7 and lead to full TLR7 deficiency (20), while other less rare variants might lead only to a partial TLR7 deficiency, and consequently impact a larger group of individuals, but exert a lower relative risk to develop severe COVID-19. Accordingly, these low effect size genetic variants in *TLR7* have been proposed as a possible explanation of the male sex bias in COVID-19 severity because of its localization on the X chromosome and well-established function in innate immunity (21). Furthermore, it is possible, but only speculative, that the addition of factors that deteriorate *TLR7* function [*e.g.* circulating 25-hydroxy vitamin D [25OHD] levels decline with age (22) and may be accompanied by a lower TLR7 expression and defective function (23)] in patients with these low effect variants may be a common cause of progression to the most severe stages of COVID-19 in male. The same speculative hypothesis could be applicable to other genes involved in immune response regulation after SARS-CoV-2 infection (4, 24).

Strong host genetic factors that confer an increased risk to develop severe COVID-19 might serve as genomic biomarkers, along with other factors, that could be used for early diagnosis and preventative measures, and could allow the identification of possible molecular targets for treatment. In this respect, there would be a strong argument to offer hemizygous *TLR7* deficient males that have not had COVID-19 direct access to early vaccination as an effective preventative measure, similar to other patients with primary immunodeficiencies. This option has indeed been offered to the hemizygous carriers in the family of patient 13. Although no data is yet available to support specific management of *TLR7* deficiency or the at-risk hemizygous carriers, early hospitalization and IFN-based therapies in inborn errors of IFN signaling form rational treatment options (18, 25).

This study has several limitations. First, no biological samples were available to functionally validate the pathogenicity of the p.(Asn215Ser) variant and therefore the functional impact of this variant remains unknown. Nevertheless, the absence of this variant in population databases, the high prediction scores for pathogenicity and the general scarcity of rare variants in *TLR7* (13) suggest this finding is unlikely due to chance. Second, the *TLR7* variant identified in patient 13 segregates in a brother who only experienced mild COVID-19. There are some explanations that may explain why a pathogenic variant in *TLR7* does not become fully penetrant, since protection against SARS-CoV-2 depends on other genetic and environmental factors (*e.g.* exposure to a lower initial viral load, previous exposure to common cold coronaviruses) (26). Moreover, TLR7 function might be specifically influenced by epidemiological factors and certain comorbidities (*e.g*. 25OHD). Thirdly, the size of this study does not permit to draw any firm conclusions on the prevalence of loss of function *TLR7* variants in males with severe COVID-19. Hence, larger cohort studies are required. Most recently, in pan-ancestry whole-exome sequencing data of 586,157 individuals, encompassing 20.592 patients who contracted COVID-19 and 1.266 of those who had severe disease, *TLR7* was the only gene in which the burden of rare variants was significantly increased among patients with severe COVID-19, using a less conservative significance threshold [OR 4.53 (2.64, 7.77)] (19). This is one of the few occasions in which a Mendelian disease gene is replicated through the use of an association study, even without stratifying for male gender or younger age.

In summary, we have identified two novel germline variants in the X chromosomal *TLR7* that likely lead to TLR7 deficiency, which is further corroborated by impaired type I and II IFN responses in the patient with the p.(Trp933Arg) variant. These findings reinforce the notion that TLR7 plays a critical role in the recognition of SARS-CoV-2 and the subsequent initiation of an early antiviral immune response that could prevent the development of severe COVID-19. More importantly, a better understanding of strong genetic factors predisposing to severe COVID-19 may help physicians to identify patients at risk. We therefore propose clinical screening criteria for the identification of *TLR7* variants in male patients with severe COVID-19. This could not only enable targeted therapeutic management of the patient, but could also offer relatives the option of pre-symptomatic testing and by extension preventative measures such as early vaccination.

## Data Availability Statement

The raw data supporting the conclusions of this article will be made available by the authors, without undue reservation.

## Ethics Statement 

The studies involving human participants were reviewed and approved by IDIBELL Research Ethics Committee (approval number PR152/20). The patients/participants provided their written informed consent to participate in this study. Written informed consent was obtained from the individual(s) for the publication of any potentially identifiable images or data included in this article.

## Author Contributions

XS, GV-P, and CM contributed equally to this work. XS, AH, and CL devised the study. GC, CM, AR-M, FS, ME, and XC provided input on the study design. AA, BH, JS-H, FV, and G-RB assisted in patient management. GV-P, AS, JV, and SR designed and performed the sequence and functional analysis. XS, GV-P, CM, AH, and CL had full access to all data and take responsibility for the integrity and the accuracy of the data. XS, GV-P, CM, AH, and CL drafted the manuscript. All authors contributed to the article and approved the submitted version.

## Funding

Contract grant sponsor: Supported by La Marató de TV3 foundation (202115-30-31), the Carlos III National Health Institute funded by FEDER funds – a way to build Europe – [PI19/00553 and CIBERONC]; the Government of Catalonia [2017SGR1282 and 2017SGR496]. AH is supported by the Solve-RD project. The Solve-RD project has received funding from the European Union’s Horizon 2020 research and innovation programme under grant agreement No. 779257. FV was supported by a ZonMW (The Netherlands Organization for Health Research and Development) Vidi grant (No. 91718351). This research was part of the Netherlands X-omics Initiative and partially funded by NWO (The Netherlands Organization for Scientific Research; project 184.034.019).

## Conflict of Interest

The authors declare that the research was conducted in the absence of any commercial or financial relationships that could be construed as a potential conflict of interest.

## Publisher’s Note

All claims expressed in this article are solely those of the authors and do not necessarily represent those of their affiliated organizations, or those of the publisher, the editors and the reviewers. Any product that may be evaluated in this article, or claim that may be made by its manufacturer, is not guaranteed or endorsed by the publisher.
