# Supplementary material for: Genetic Screening for TLR7 Variants in Young and Previously Healthy Men With Severe COVID-19
Source: Front Immunol. 2021 Jul 23;12:719115. doi: 10.3389/fimmu.2021.719115 (PMC8343010; doi:10.3389/fimmu.2021.719115)
Supplement: Supplementary file 1 [file DataSheet_1.pdf]

Supplementary Materials

Table of Contents

Supplemental Figures and Tables

Figure S1..………………………………………………………………………………… 2
Figure S2..…………………………………………………………………………….... 3
Table S1…………………………….. 4
References………………………………………………………….………………………… 5

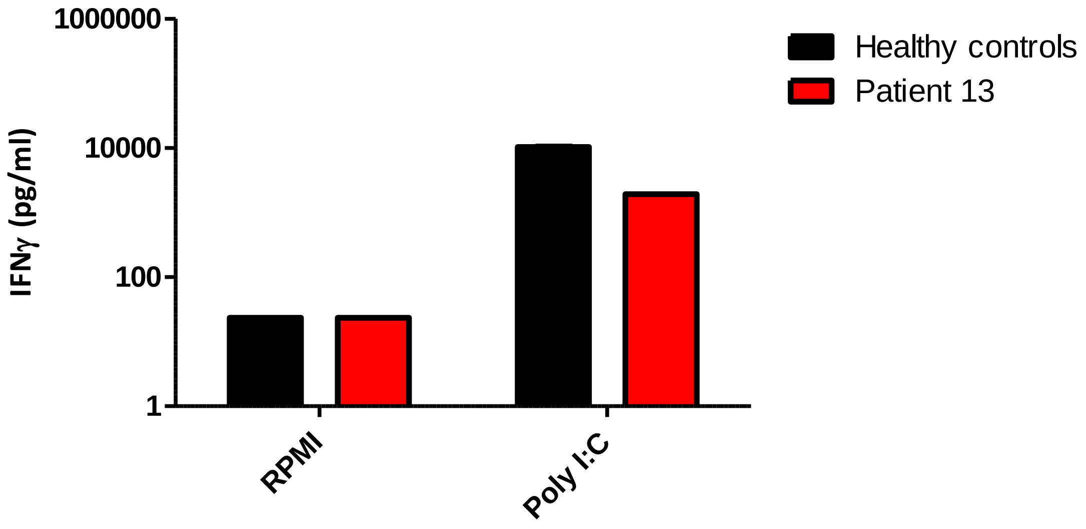

Figure S1. Production of IFN-γ in peripheral blood mononuclear cells from patient 13 and two healthy male controls after stimulation with the TLR3 agonist Poly I:C (25μg/mL) or the negative medium control RPMI for 7 days. Absolute IFN-γ values are represented on a log-based scale.

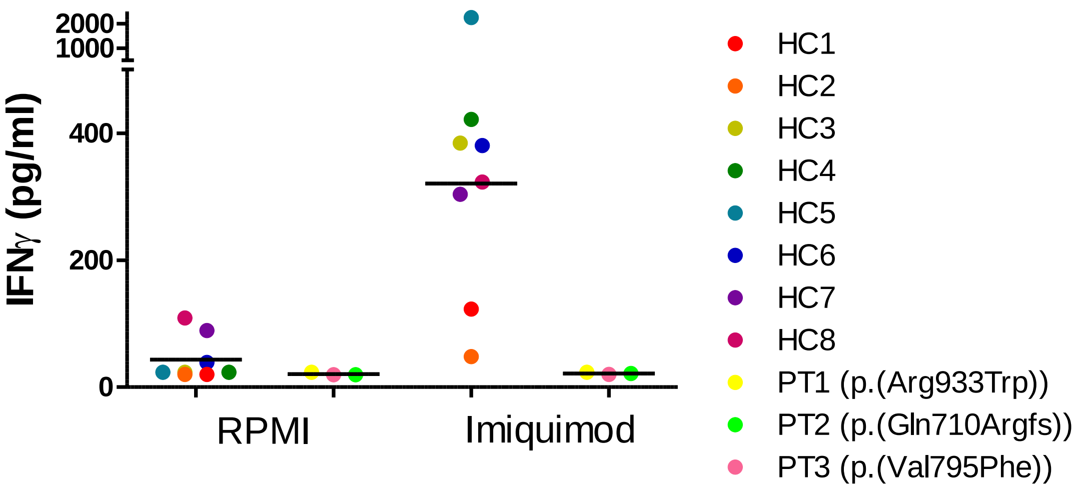

Figure S2. Production of IFN-γ in peripheral blood mononuclear cells isolated from healthy male controls or TLR7 patients in independent experiments after 7 day stimulation with the TLR7 agonist imiquimod (5μg/mL) or the negative medium control RPMI. Data from PT2 and PT3 correspond to absolute values as documented for patients F1.II.1 and F2.II.1, respectively, in the previous paper of van der Made et al. JAMA 2020(1). Horizontal lines represent grand means.

Table S1. In silico results from the mutation interpretation software Alamut Visual v.2.5.0 of the *TLR7* variants identified in this work

***TLR7* cDNA change**	**c.644A>G**	**c.2797T>C**
Protein predicted change	p.Asn215Ser	p.Trp933Arg
phyloP	Highly conserved nucleotide (score:4.64)	Moderately conserved nucleotide (score:3.27)
Orthologues Alignments	Highly conserved aminoacid, up to zebrafish (11 species)	Highly conserved aminoacid, up to fruitfly (12 species)
Grantham	Small:46	Moderate:101
Align GVGD	C45	C25
SIFT	Deleterious (score:0)	Deleterious (score: 0)
MutationTaster	Disease causing (probability:1)	Disease causing (probability: 1)
PolyPhen HumDiv	Possibly damaging (score:0.78)	Probably damaging (score:1)
PolyPhen HumVar	Possibly damaging (score:0.54)	Probably damaging (score:0.99)

References

1.- van der Made CI, Simons A, Schuurs-Hoeijmakers J, van den Heuvel G, Mantere T, Kersten S, et al. Presence of Genetic Variants Among Young Men With Severe COVID-19. *JAMA* (2020) 324(7):663-73. doi: 10.1001/jama.2020.13719.
